# Cannabis in Homes with Children: A Survey on Use, Storage, and Attitudes

**DOI:** 10.5811/westjem.2021.5.49057

**Published:** 2021-09-02

**Authors:** Alex Gimelli, Anusha Deshpande, Julia N. Magana, Aimee Moulin

**Affiliations:** * UC Davis Medical Center, Department of Emergency Medicine, Sacramento, California; † Scripps Mercy Hospital, Department of Internal Medicine, San Diego, California

## Abstract

**Introduction:**

The recent legalization of cannabis in California has the potential to affect cannabis prevalence in households with children. This eventuality, combined with suboptimal cannabis storage practices, could lead to adverse effects such as unintentional pediatric ingestion, which occurred in Colorado after legalization. Our objective was to assess prevalence and storage practices of cannabis in households with children, and attitudes on use and storage education in a state that has legalized cannabis.

**Methods:**

We administered electronic surveys to 401 adults in a pediatric emergency department in California. Participants were excluded if they were not English- or Spanish-speaking or did not live in a household with children <18 years old. They answered questions regarding cannabis use, storage, and attitudes on cannabis storage education. We used convenience sampling and analyzed data using descriptive statistics.

**Results:**

Research assistants approached 558 participants of whom 401 completed the survey. Three participants did not respond regarding past or current cannabis use, and 14.5% (58/401) reported cannabis use in their home in the prior six months. Both users and non-users rated safe storage of high importance in homes with children. Only 44.8% of home users (26/58) reported that their cannabis was both locked and hidden. Among home users, the most common source of storage advice was friends and family (21/58, 36.2%), and 45% of home users (26/58) received no storage information whatsoever. Most cannabis users (53/67, 79.1%) and non-users (241/330, 73%) reported that they would feel comfortable receiving cannabis education from their primary care provider.

**Conclusion:**

Cannabis is used and stored in homes with children; however, safe storage is not clearly defined in California, and storage education is lacking. Healthcare providers in primary care and the emergency department may play an important role in educating the public about cannabis use and safe storage.

## INTRODUCTION

Legalization of cannabis use is increasingly widespread across the United States, but the ramifications are unknown. In 2016 California approved Proposition 64 legalizing recreational cannabis. Unintentional pediatric ingestion is one possible ramification, as occurred in Colorado after legalization in 2009. Regional poison center cases involving marijuana increased by an average 34% per year from 2009 to 2015 in Colorado. During that time, 34% of cases involved self-reported cases of poor product storage.^1^ Colorado instituted safe storage guidelines to mitigate adverse effects in children from acute cannabis intoxication.^2^ Children who unintentionally ingest cannabis can present with lethargy, ataxia, tachycardia, mydriasis, and hypotonia, which can lead to preventable emergency department (ED) visits, invasive workups, and hospital admissions.^3^,^4^

Despite the institution of safe storage guidelines in Colorado, a recent study found continuing suboptimal storage practices in that state.^5^ This trend was mirrored in the use of medical marijuana, as oncology patients and their caregivers reported suboptimal storage practices and had received little storage education from healthcare providers.^6^ The 2016 California legislation did not include regulations on the safe storage and disposal of cannabis products, creating a potential for similarly unsafe storage practices. The purpose of this study, which was based on a community presenting to a pediatric ED, was to assess the prevalence of cannabis and how it is stored in the home and, secondly, to assess attitudes regarding use of cannabis and storage education among Californians who live in households with children.

## METHODS

We conducted a cross-sectional survey with a goal enrollment of 400 adult visitors in an academic pediatric ED in California from June 8–August 16, 2018. During this time, a convenience sample between the hours of 8 am –10 pm was conducted daily in which all adult visitors were screened for eligibility and subsequently approached. The survey was generated and finalized by the investigators and research assistants (RA) based on similar studies found during literature review. The survey contained 42 yes-no or Likert-scale questions regarding cannabis use and storage, and education on cannabis storage. Eligible participants were ≥18 years old and lived in a household with children <18 years old. Participants were excluded if they did not speak English or Spanish, or if the patient was critically ill. Only one survey was administered per household. All participants were notified that their responses were not shared with law enforcement or their care team, and they completed the survey in the absence of a RA.

English-speaking participants filled out the survey electronically and submitted their responses directly into Research Electronic Data Capture (REDCap Consortium, Vanderbilt University, Nashville, TN). Spanish-speaking participants filled out a Spanish-language survey on paper, which was subsequently placed in a lockbox after which these de-identified surveys were uploaded to REDCap weekly. We used descriptive statistics to analyze data. Subjects who were screened but excluded were not tracked during this study. The UC Davis Institutional Review Board approved this study.

## RESULTS

Research assistants approached 558 visitors who met inclusion criteria, and 401 (71.9%) consented to participate (Table 1). Seventeen percent of participants (67/401) reported cannabis use in the last six months, and 14.5% (58/401) reported cannabis use in their home. The 82.3% (330/401) of respondents who denied use of cannabis within the last six months are referred to as non-users for the purposes of data analysis. Four respondents did not disclose their cannabis use within the last six months. Inhaled marijuana (46/57, 81%) and edibles (22/57, 39%) were reportedly the most common forms of cannabis used in the home (Table 2).

On a scale of 1=Extremely unsafe to 10=Extremely safe, cannabis users had a mean safety score regarding use of cannabis around children at 6.72/10, whereas non-users felt cannabis use to be less safe around children with a mean score of 3.28/10. Over half of home users (32/58, 55.2%) reported keeping their cannabis in a locked container, and only 44.8% of home users (26/58) reported keeping their cannabis both locked and hidden.

Among home users, the most common source of storage advice was friends and family (21/58, 36.2%), and 52% of these individuals (11/21) cited this as their lone source. Only 16% of home users (9/58) received safe storage information from a dispensary, and 45% of home users (26/58) received no storage information whatsoever or claimed they did not know of sources of storage information because the cannabis did not belong to them. Only 7% of home users received education on use and storage from their primary care provider (PCP) (4/58). Most of both cannabis users (53/67, 79.1%) and non-users (241/330, 73%) were comfortable receiving education about cannabis from their PCP. Most cannabis users (42/67, 62.7%) and non-users (221/330, 67%) felt that PCPs should screen and educate their patients on cannabis use/safe storage. Similarly, both cannabis users (38/67, 56.7%) and non-users (188/330, 57%) felt that dispensaries should educate the public on cannabis use/safe storage.

## DISCUSSION

A 2017 national survey indicated that as many as 11.5% of California adults reported regular cannabis use. Of adults surveyed, 14.5% reported cannabis use in a home with children. Since the legalization of cannabis in California, there has been a steady rise in prevalence of use,^7^ likely due to increased availability. In our sample, users perceived cannabis to be significantly safer for both adult use and possession inside a home with children, as compared with non-users. Further study is warranted to investigate how the public perceives the risk of cannabis as more time passes since legalization.

Currently, little research exists on cannabis storage in homes with children, and there is no research that describes sources of storage information. Both users and non-users strongly felt that safe storage was important despite poor compliance with safe storage practices. Our results suggest a lack of educational sources regarding safe storage despite 23 years of medical cannabis use in California.^2^ The Public Health Department of Colorado set guidelines including locking, hiding, and using child-resistant packaging, yet California does not currently define safe storage. Providing guidelines at a local or state level may provide a reference for cannabis users as well as healthcare providers. Based on participant responses, cannabis dispensaries may also serve as another point for the distribution of safe storage information.

With the increasing prevalence of cannabis use in California,^7^,^8^ downstream effects on the pediatric population should be further investigated. Healthcare providers in primary care, pediatrics, and the ED should be prepared to screen and educate families on cannabis use and the importance of safe storage in homes with children.

## LIMITATIONS

Limitations of this study include the use of a single site for data collection. We used a convenience sample within the hours of 8 am – 10 pm, which may have incurred selection bias. The survey tool used was not rigorously validated by respondents or external experts. The study relied on self-reporting, and stigma regarding cannabis use may have skewed self-reporting and enrollment. Furthermore, the use of paper surveys for Spanish speakers due to the lack of an electronic survey may have altered results for the Spanish-speaking respondents. No ethnic and socioeconomic data was collected, and this may limit the application of this sample to the public.

## CONCLUSION

Safe cannabis storage is not clearly defined in California, and there is a lack of safe storage education. In our sample we found that many children are exposed to cannabis use in their homes, and most cannabis users do not keep their cannabis both locked and hidden. Most cannabis users and non-users alike felt comfortable discussing cannabis storage safety with their providers. Healthcare providers in primary care and the ED may play an important role in educating the public about cannabis use and safe cannabis storage.

## Figures and Tables

**Table 1:** Participant demographics and history of cannabis use.

	n =	% of Subgroup	Mean Age	SD	Mean # Children	SD
Total participants	401	100%	35.3	9.9	2.4	1.4
Male	109	27.2%	37.2	11.0	2.3	1.7
Female	283	70.1%	34.8	9.2	2.2	1.2
Other gender	7	1.7%	34.8	10.7	2.6	1.4
Undisclosed gender	2*	0.2%	21*	0*	2*	0*
Tried at least once	197	49.1%	34.7	9.9	2.2	1.3
Males	54	49.5%	35.9	10.8	2.1	1.4
Females	137	48.4%	34.3	9.4	2.3	1.2
Other	5	71.4%	33.6	11.5	2.6	1.4
Undisclosed gender*	1	50%	21	0	2	0
Use in last 6 months	67	16.7	32.3	10.4	2.1	1.3
Males	23	21.1	33.3	12.2	1.7	1.4
Females	42	14.8	32.1	9.4	2.3	1.3
Other	1	14.3	28	0	5	0
Undisclosed gender*	1	50%	21	0	2	0
Use in home w/child	58	14.5	32.9	10.3	2.1	1.3
Males	17	15.6	31.4	8.8	1.6	1.3
Females	40	14.1	33.9	10.7	2.3	1.3
Other	0					
Undisclosed gender*	1	50%	21	0	2	0

* Two participants did not disclose gender. One did not disclose any demographic information.

*SD*, standard deviation.

**Table 2:** Cannabis users’ products, storage practices, and sources of storage education.

	n=58	%
Cannabis product used in homes		
Edible cannabis products	22	37.9
Smoked marijuana	46	79.3
Hashish	4	6.9
Hashish oil	10	17.2
Wax	18	31
Other	4	6.9
Cannabis storage		
Locked	32	55.2
Hidden	42	72.4
Locked and hidden	26	44.8
Plain sight	4	6.9
Medicine cabinet	6	10.3
Within reach of children <12	4	6.9
Out of reach of children <12	23	40
Common areas	2	3.4
I don’t know where the cannabis is kept	5	8.6
Other locations	6	10.3
Sources of storage information among cannabis owners^*^		
“I don’t know. It isn’t mine.”	17	29.3
Primary care provider (pediatrician, your primary doctor)	4	6.9
Guidelines	7	29.5
Cannabis dispensary	9	15.5
Friends/family	21	36.2
Online advice	6	10.3
Other unspecified	2	4.9
None	9	15.5
